# The use of oral HIV pre-exposure prophylaxis among people who inject drugs: barriers, and recommendations

**DOI:** 10.3389/fpubh.2023.1265063

**Published:** 2023-09-05

**Authors:** Tafadzwa Dzinamarira, Enos Moyo

**Affiliations:** ^1^School of Health Systems and Public Health, University of Pretoria, Pretoria, South Africa; ^2^ICAP at Columbia University, Lusaka, Zambia; ^3^School of Nursing and Public Health, College of Health Sciences, University of Kwa-Zulu Natal, Durban, South Africa

**Keywords:** people who inject drugs, human immunodeficiency virus, oral pre-exposure prophylaxis, barriers, recommendations

## 1. Introduction

Globally, it is estimated that 296 million people used illicit drugs in 2021, which was a 23% increase from the number reported in 2011. Of the drug users in 2021, about 13 million were injection drug users (1). The burden of injection drug use is different in different regions of the world. About 1.3% of the adult population in Eastern Europe inject drugs while 1% in North America also inject drugs (1). People who inject drugs (PWID) are 35 times more likely to acquire HIV compared to those who do not inject drugs and about 12% of PWID globally live with HIV (1). The distribution of HIV among PWID globally is heterogeneous. Regions with the highest prevalence of HIV among PWID are Southwest Asia, with 29.3%, and Eastern Europe with 25.4% (1). The risk factors for HIV among PWID include the sharing of drug injection equipment such as syringes and needles, inconsistent use of condoms, the use of unsterilized injection equipment, having multiple sexual partners, and exchanging sex for drugs and money (2, 3). Women who inject drugs (WWID) are 1.2 times more likely than males to have HIV, even though men are five times more likely to inject drugs than women (1). This gender disparity has been attributed to sex work among WWIDs, their vulnerability to abuse by law enforcement officers, and physical violence or rape (1).

Various HIV preventive strategies can be applied to PWID. These strategies include harm reduction services, behavioral intervention, opioid agonist therapy, and pre-exposure prophylaxis (PrEP) (4). Common harm reduction services include needle exchange programs and over-the-counter syringe sales (5). The transmission of HIV among PWID must be decreased using a combination of these strategies. Harm reduction methods, opioid agonist therapy, and behavioral interventions are difficult to implement due to structural barriers such as the criminalization of drug use in many countries, as well as stigma and discrimination. According to a study conducted in the United States of America, < 20% of patients with opioid use disorder are on opioid agonist therapy, and < 30% of these are retained in care at 6 months after initiation (4). The use of oral HIV PrEP is an additional method that can help reduce HIV transmission among PWID. In the Bangkok Tenofovir Study, oral PrEP was proven to be efficacious among PWID (6). According to the study, those taking oral Tenofovir (TDF) had a 48.9% lower risk of contracting HIV than those receiving a placebo (6). Additionally, subsequent analysis showed that participants with an adherence of 97.5% or higher experienced an 83.5% decrease in new HIV infections (7).

Several studies have revealed that PrEP is acceptable among PWID (8–11). A study conducted in Tanzania revealed that 71.4% of the participants were willing to use oral PrEP if it was made available to them (8). Factors that were associated with the willingness to use oral PrEP in the study were the risk of HIV infection, the frequency of condomless sex, the number of sexual partners, and being in an HIV prevention program (8). Another study conducted in India revealed a 52.4% willingness to use oral PrEP among participants (9). A study conducted in the United States of America (USA) revealed a 59% willingness to use oral PrEP among participants (10) while another revealed 63% (11). Although the willingness to use oral PrEP was high in several studies, the actual uptake has been low. A study conducted in the USA revealed that although there was a 59% willingness to use oral PrEP among participants, only two per cent were taking oral PrEP at the time (10). Another study also conducted in the USA revealed that only 0.15% of PWID who had commercial insurance were taking oral PrEP (12). In this article, we aimed to (i) summarize evidence on accessibility, and uptake of oral PrEP among PWID, (ii) discuss the barriers to oral PrEP use among PWID and (iii) present a perspective on the strategies that can be used to overcome these barriers.

## 2. Barriers to oral PrEP use among PWID

PWID are unable to access oral PrEP globally due to several barriers. These barriers can be classified as individual-level barriers, community and health system-level barriers, and structural barriers.

### 2.1. Individual-level barriers

Individual-level barriers to oral PrEP uptake among PWID include low PrEP knowledge, low HIV risk perception, concerns about oral PrEP side effects, and competing health priorities and needs due to drug use and dependence (13). The low PrEP knowledge among PWID can be attributed to the absence of PrEP education programs for them. Reaching PWID may be a challenge since they usually remain underground to avoid being arrested (14). PWID are often intoxicated, making it difficult for them to comprehend their risk of being infected with HIV. Even when not intoxicated, some PWID may perceive that they have a low risk of HIV due to a lack of information about HIV (15). Among those who know about PrEP, some may be unwilling to use PrEP due to concerns about the side effects of the drugs. This may emanate from misinformation or myths about PrEP drugs (13). PWID have immediate priorities such as cash to get their drugs and where to sleep if they are homeless. Therefore, if access to oral PrEP requires transportation, PWID may be unwilling to use their money for transport instead of their injection drugs (16). Furthermore, because PWID may be homeless, even if they get oral PrEP, the drugs can be damaged by rain or snow, or they can be stolen. Intoxication may also result in PWID forgetting to take their medication and to go for their follow-up appointments (16).

### 2.2. Community and health system-level barriers

PWID usually face stigma and discrimination from their families, communities, and healthcare workers when they go to healthcare facilities to seek healthcare services. A study conducted in Tanzania revealed that about 71% of participants had experienced higher levels of stigma (17). The stigma experienced by PWID can be enacted, anticipated, or internalized. Enacted stigma is defined as experiences of discrimination or prejudice related to drug use felt by PWID while anticipated stigma is the expectation of future discrimination or prejudice felt by PWID. Internalized stigma is the acceptance of negative views and self-devaluation as a result of drug use (18). When PWID complain of specific health issues, certain healthcare workers (HCWs) may have dismissive attitudes toward them and attribute the issues to drug usage. Additionally, HCWs may be judgmental of PWID or use cruel language, which can lead to PWID losing their sense of dignity and self-worth (19). Stigmatizing experiences influence healthcare-seeking behavior by PWID. The fear of being stigmatized or discriminated against may lead to PWID avoiding HCWs and healthcare facilities. As a result of the fear of stigma and/or discrimination, or previous stigma and/or discrimination, PWID may not seek HIV services such as HIV testing and PrEP (20). Furthermore, PWID may fail to access PrEP when they go for other healthcare services because the other services may not offer PrEP services (16).

### 2.3. Structural barriers

Distance to healthcare facilities, involvement in the criminal justice system, and a lack of identification documents are structural barriers that may impede PWID from accessing oral PrEP (13). PWID who stay far away from healthcare facilities may find it difficult to go to healthcare facilities due to a lack of money for transport (16). Due to homelessness, some PWID may find it difficult to always have identification documents, which may be required at healthcare facilities before they can access healthcare, including HIV services such as the provision of PrEP. Identification documents may be destroyed by rain or fire or may be lost. When PWID do not have identification documents, they may avoid going to healthcare facilities to seek PrEP (13). Furthermore, some PWID may not seek PrEP services out of fear that they may be handed over to law enforcement agents for incarceration (21).

## 3. Strategies to address the barriers to oral PrEP use among PWID

To address the barriers to oral PrEP use among PWID, we recommend several strategies. The strategies include PrEP education programs for PWID, rehabilitation for drug abuse, reducing stigma and discrimination against PWID, integration of PrEP services into other healthcare services, and the use of differentiated service delivery models and new long-acting products.

### 3.1. PrEP education programs for PWID

To improve PrEP knowledge among PWID, we recommend educational programs that target them. These programs should include the dissemination of PrEP education in various media platforms such as social and mass media, as well as information leaflets and billboards (22). HCWs should provide PrEP knowledge to PWID when they are being attended for other health problems, including drug addiction rehabilitation services (23). PrEP education programs should also involve peer educators, especially those who have been successfully rehabilitated since they may be trusted by PWID (22). We recommend that the provision of PrEP knowledge should include information about the common side effects of oral PrEP, as well as address the misconceptions and myths about oral PrEP. In addition, we recommend that the programs should emphasize that the side effects are usually transient.

### 3.2. Rehabilitation for drug abuse

PWID may forget to take their oral PrEP because of intoxication. Moreover, they may prioritize injection drugs instead of oral PrEP when they get money. To address these problems, we recommend emphasizing the rehabilitation of PWID. Rehabilitation services for PWID should be accessible, affordable, and they should be offered in a non-judgmental way (5). Privacy and confidentiality of PWID who use these services should be maintained at all times as this will ensure trust between PWID and HCWs, including the healthcare system.

### 3.3. Reducing stigma and discrimination

To reduce stigma and discrimination against PWID taking oral PrEP in the community, we recommend that communities be informed about the importance of oral PrEP and tolerance toward PWID, as well as being engaged in oral PrEP programs (24). We recommend the information be disseminated through various media channels such as social and mass media, as well as community events such as sports competitions. To reduce stigma and discrimination against PWID at healthcare facilities, we recommend providing HCWs with knowledge about the needs of PWID and training them on client relations so that they can improve their attitudes toward PWID. Training of HCWs on the needs of PWID can be conducted through in-person, face-to-face contact, educational lectures, video-based contact, or in-person interactive games (25). We also recommend that PWID be provided with communication channels to raise their complaints to the management of healthcare facilities anonymously. Such channels may include toll-free lines or complaints boxes.

### 3.4. Integration of oral PrEP services into other healthcare services

PWID may seek healthcare services such as opioid agonists as part of their rehabilitation or treatment of infections at injection sites. We recommend that these encounters with PWID be utilized to offer them oral PrEP. Integrating oral PrEP services into other services may be cost-effective, considering that some of the infrastructure will already be in place. Furthermore, fewer HCWs may be required compared to when the services are offered separately (8). In addition, providing oral PrEP in a range of routine settings may help reduce the stigma associated with specialized clinics and HIV services (26). However, the HCWs may need to be trained on the provision of oral PrEP services since some may not be familiar with the services.

### 3.5. Use of differentiated service delivery models and new long-acting products

PWID may avoid going to healthcare facilities because of previous experiences of being stigmatized and discriminated against. To address this barrier, we recommend the community-based provision of oral PrEP. Community-based services may include clinics managed by non-governmental organizations, mobile and home-based services, telehealth linked with laboratories and pharmacies, and peer-led outreach services to cater to the homeless (27). Differentiated service delivery models can also help reduce the workload of HCWs at healthcare facilities, especially in low-to-middle-income countries that have understaffed healthcare facilities (28). We also recommend the use of long-acting products for PrEP such as long-acting injectable Cabotegravir and Dapivirine vaginal ring as they may help solve the problems of forgetfulness and having to return to the healthcare facilities frequently (26).

## 4. Conclusion

Oral HIV PrEP is an effective way to reduce the risk of HIV transmission among PWID. However, uptake of PrEP among PWID has been low. This is due to several factors, including individual-level barriers, such as lack of knowledge about PrEP; community-level barriers, such as stigma and discrimination against PWID; health system-level barriers, such as limited access to PrEP services; and structural barriers, such as poverty and lack of access to healthcare. To address these barriers, we recommend a number of strategies, including PrEP education programs for PWID, rehabilitation for drug abuse, reducing stigma and discrimination against PWID, integrating PrEP services into other healthcare services, and using differentiated service delivery models and new long-acting products.

## Author contributions

TD: Conceptualization, Writing—original draft. EM: Conceptualization, Writing—review and editing.

## References

[1] United Nations Office of Drugs and Crime. World Drug Report 2023. (2023). Available online at: https://www.unodc.org/unodc/en/data-and-analysis/world-drug-report-2023.html (accessed July 12, 2023).

[2] Pachuau L Tannous C Dhami M Agho K HIV. among people who inject drugs in India: a systematic review. BMC Public Health. (2022) 22:1529. 10.1186/s12889-022-13922-2 35948967 PMC9367073

[3] Hogan S Page A Ogbo F Dixit S Rajbhandari RM Rawal B. Trends and determinants of HIV transmission among men who inject drugs in the Pokhara Valley, Nepal: analysis of cross-sectional studies. BMC Public Health. (2021) 21:269. 10.1186/2Fs12889-021-10331-9 33530983 PMC7856790

[4] Strathdee SA Kuo I El-Bassel N Hodder S Smith LR Springer SA. Preventing HIV outbreaks among people who inject drugs in the United States: plus ça change, plus ça même chose. AIDS. (2020) 34:1997–2005. 10.1097/2FQAD.0000000000002673 32826391 PMC7606503

[5] Fernandes RM Cary M Duarte G Jesus G Alarcão J Torre C. Effectiveness of needle and syringe Programmes in people who inject drugs—An overview of systematic reviews. BMC Public Health. (2017) 17:309. 10.1186/s12889-017-4210-2 28399843 PMC5387338

[6] Choopanya K Martin M Suntharasamai P Sangkum U Mock PA Leethochawalit M. Antiretroviral prophylaxis for HIV infection in injecting drug users in Bangkok, Thailand (the Bangkok Tenofovir Study): a randomised, double-blind, placebo-controlled phase 3 trial. Lancet. (2013) 381:2083–90. 10.1016/S0140-6736(13)61127-7 23769234

[7] Martin M Vanichseni S Suntharasamai P Sangkum U Mock PA Leethochawalit M. The impact of adherence to preexposure prophylaxis on the risk of HIV infection among people who inject drugs. AIDS. (2015) 29:819–24. 10.1097/qad.0000000000000613 25985403

[8] Iseselo M Tarimo E Sandstrom E Kulane A. Awareness and willingness to use HIV oral pre-exposure prophylaxis among people who inject drugs in Dar es Salaam, Tanzania: a cross-sectional survey. PLoS Glob Public Health. (2022) 2:e0000776. 10.1371/journal.pgph.0000776 36962766 PMC10121179

[9] Belludi A McFall AM Solomon SS Celentano DD Mehta SH Srikrishnan AK. Awareness of and willingness to use pre-exposure prophylaxis (PrEP) among people who inject drugs and men who have sex with men in India: Results from a multi-city cross-sectional survey. PLoS ONE. (2021) 16:e0247352. 10.1371/journal.pone.0247352 33630909 PMC7906475

[10] Walters SM Kral AH Simpson KA Wenger L Bluthenthal RN HIV. Pre-exposure prophylaxis prevention awareness, willingness, and perceived barriers among people who inject drugs in Los Angeles and San Francisco, CA, 2016–2018. Subst Use Misuse. (2020) 55:2409–19. 10.1080/10826084.2020.1823419 32962490 PMC7665852

[11] Sherman SG Schneider KE Park JN Allen ST Hunt D Chaulk CP. PrEP awareness, eligibility, and interest among people who inject drugs in Baltimore, Maryland. Drug Alcohol Depend. (2019) 195:148–55. 10.1016/j.drugalcdep.2018.08.014 30639794 PMC6436943

[12] Streed CG Morgan JR Gai MJ Larochelle MR Paasche-Orlow MK Taylor JL. Prevalence of HIV preexposure prophylaxis prescribing among persons with commercial insurance and likely injection drug use. JAMA Netw Open. (2022) 5:e2221346. 10.1001/jamanetworkopen.2022.21346 35819784 PMC9277489

[13] Biello KB Bazzi AR Mimiaga MJ Biancarelli DL Edeza A Salhaney P. Perspectives on HIV pre-exposure prophylaxis (PrEP) utilization and related intervention needs among people who inject drugs. Harm Reduct J. (2018) 15:15. 10.1186/s12954-018-0263-5 30419926 PMC6233595

[14] Surratt HL Yeager HJ Adu A Gonzalez EA Nelson EO Walker T. Pre-exposure prophylaxis barriers, facilitators and unmet need among rural people who inject drugs: a qualitative examination of syringe service program client perspectives. Front Psychiatry. (2022) 13:314. 10.3389/fpsyt.2022.905314 35706473 PMC9189386

[15] Mburu G Limmer M Holland P HIV. risk behaviours among women who inject drugs in coastal Kenya: findings from secondary analysis of qualitative data. Harm Reduct J. (2019) 16:10. 10.1186/s12954-019-0281-y 30728012 PMC6364406

[16] Allen ST O'Rourke A White RH Smith KC Weir B Lucas GM. Barriers and facilitators to PrEP use among people who inject drugs in rural appalachia: a qualitative study. AIDS Behav. (2020) 24:1942–50. 10.1007/2Fs10461-019-02767-3 31853771 PMC7228835

[17] Likindikoki SL Meyrowitsch DW Mizinduko MM Ishungisa AM Tersbøl BP Leyna GH. Socio-cognitive factors influencing access to HIV prevention services among people who inject drugs in Dar es Salaam, Tanzania: an integrated bio-behavioural survey. PLoS ONE. (2022) 17:e0261500. 10.1371/journal.pone.0261500 35089928 PMC8797198

[18] Muncan B Walters S Ezell J Ompad D. “They look at us like junkies”: influences of drug use stigma on the healthcare engagement of people who inject drugs in New York City. Harm Reduct J. (2020) 17:53. 10.1186/s12954-020-00399-8 32736624 PMC7393740

[19] Paquette C Syvertsen J Pollini R. Stigma at every turn: Health services experiences among people who inject drugs. Int J Drug Policy. (2018) 57:104–10. 10.1016/j.drugpo.2018.04.004 29715589 PMC5994194

[20] Fong C Mateu-Gelabert P Ciervo C Eckhardt B Aponte-Melendez Y Kapadia S. Medical provider stigma experienced by people who use drugs (MPS-PWUD): development and validation of a scale among people who currently inject drugs in New York City. Drug Alcohol Depend. (2021) 221:108589. 10.1016/2Fj.drugalcdep.2021.108589 33621804 PMC8029599

[21] Dengo-Baloi L Boothe M Semá Baltazar C Sathane I Langa DC Condula M. Access to and use of health and social services among people who inject drugs in two urban areas of Mozambique, 2014: qualitative results from a formative assessment. BMC Public Health. (2020) 20:975. 10.1186/s12889-020-09068-8 32571365 PMC7310000

[22] Rosen JG Zhang L Pelaez D Park JN Glick JL A. Capacity-Strengthening Intervention to Support HIV Pre-exposure Prophylaxis (PrEP) awareness-building and promotion by frontline harm reduction workers in Baltimore, Maryland: a mixed methods evaluation. AIDS Behav. (2023) 27:2440–53. 10.1007/s10461-022-03971-4 36596866 PMC9810241

[23] Walters SM Frank D Van Ham B Jaiswal J Muncan B Earnshaw V. PrEP care continuum engagement among persons who inject drugs: rural and urban differences in stigma and social infrastructure. AIDS Behav. (2022) 26:1308–20. 10.1007/2Fs10461-021-03488-2 34626265 PMC8501360

[24] Dutta M. Disseminating HIV pre-exposure prophylaxis information in underserved communities. Am J Prev Med. (2013) 1:S133–6. 10.1016/j.amepre.2012.09.030 23253754

[25] Muwonge TR Nsubuga R Ware NC Wyatt MA Pisarski E Kamusiime B. Health care worker perspectives of HIV pre-exposure prophylaxis service delivery in Central Uganda. Front Public Health. (2022) 10:658826. 10.3389/2Ffpubh.2022.658826 35444979 PMC9013815

[26] Henderson M Schmidt HM Chitembo L Peralta H Alaama AS Johnson C. The Future of pre-exposure prophylaxis (PrEP) for HIV prevention: a global qualitative consultation on provider perspectives on new products and differentiated service delivery. AIDS Behav. (2023) 2023:1. 10.1007/s10461-023-04093-1 37351685 PMC10589125

[27] Shaw G Schaefer R Schmidt HM Madden A Chang J Mozalevskis. Pre-exposure prophylaxis (PrEP) for HIV prevention among people who inject drugs: a global mapping of service delivery. Harm Reduct J. (2023) 20:16. 10.1186/s12954-023-00729-6 36782321 PMC9924874

[28] Nishimoto L Ikani P Achanya J Idowu A Olisa AL Walker CF. Expanding access to oral preexposure prophylaxis for people who inject drugs in Bayelsa and Niger States, Nigeria. Glob Health Sci Pract. (2023) 11:e2200370. 10.9745/GHSP-D-22-00370 36853631 PMC9972381

